# Draft Genome Sequence of the Fish Pathogen Flavobacterium columnare Genomovar III Strain PH-97028 (=CIP 109753)

**DOI:** 10.1128/genomeA.00222-18

**Published:** 2018-04-05

**Authors:** Alexis Criscuolo, Olivier Chesneau, Dominique Clermont, Chantal Bizet

**Affiliations:** a Institut Pasteur, Bioinformatics and Biostatistics Hub-C3BI, USR 3756 IP CNRS, Paris, France; b Microbiology Department, Institut Pasteur, Collection de l’Institut Pasteur, Paris, France

## Abstract

Flavobacterium columnare strain PH-97028 (=CIP 109753) is a genomovar III reference strain that was isolated from a diseased Ayu fish in Japan. We report here the analysis of the first available genomovar III sequence of this species to aid in identification, epidemiological tracking, and virulence studies.

## GENOME ANNOUNCEMENT

There is a high genetic heterogeneity within the species Flavobacterium columnare, a yellow-colored, Gram-negative, rod-shaped bacterium that is known as the etiologic agent of columnaris disease in freshwater fish. Despite diverse hosts and disease outbreaks worldwide, F. columnare isolates cannot be phenotypically distinguished, and subcategorization relies on three clades or genotypes whose rRNA gene is the support (1). As of February 2018, only 15 genomes of F. columnare isolates are publicly available, among which, representative ones belong to only genomovar I (e.g., strains ATCC 49512 and Pf1) or II (e.g., strains C#2 and 94-081) (2). Here, we report the first genome assembly of a representative strain from F. columnare genomovar III, strain PH-97028 (=CIP 109753), for which no functional data have been analyzed thus far.

A single colony of strain PH-97028 was grown overnight in 10 ml of CIP medium 469 (10 g peptone, 10 g yeast extract, 5 g sodium chloride, and 1 liter distilled water) at 30°C with shaking for 24 h. Total cellular DNA was obtained from 2 ml of culture using the Wizard genomic DNA purification kit (Promega, Madison, WI, USA). Libraries were constructed using the Nextera XT DNA library preparation kit (Illumina, Inc., San Diego, CA, USA) and sequenced on a NextSeq 500 instrument using a 2 × 150-bp paired-end protocol, leading to 1,409,297 read pairs (>120× average sequencing depth, ∼250 bp insert size).

Sequenced reads were clipped and trimmed with AlienTrimmer (3) and corrected with Musket (4). The remaining processed reads were assembled and scaffolded with SPAdes (5). The draft genome has a total size of 3,255,527 bp, represented in 204 scaffolds, with an *N*_50_ value of 51,970 bp. Its G+C content of 30.66% is slightly lower than the G+C content of genomovar I and II genomes (e.g., 31.5% and 30.9%, respectively). Scaffold sequences were processed with Prokka (6) for gene prediction and annotation, which led to the detection of 2,727 coding genes and 41 tRNAs.

Functional genome analysis of strain PH-97028 revealed the presence of orthologs of the F. johnsoniae genes *gldK*, *gldL*, *gldM*, *gldN*, *sprA*, *sprE*, *sprT*, *porU*, and *porV*. The proteins encoded by these genes are components of the type IX secretion system machinery that was shown to be involved in gliding motility and virulence properties for several members of the phylum Bacteroidetes (7). In addition to numerous toxins and mobile elements, and contrary to the F. columnare genomovar I and II genomes, the genome of strain PH-97028 would encode a group II intron reverse transcriptase/maturase and a complete HemTUV ATP-binding cassette system.

The two-way average nucleotide identity values between F. columnare strain PH-97028 (genomovar III) and F. columnare representative strains from genomovars I and II vary from 85.09% to 85.60%, respectively, confirming that strain PH-97028 belongs to a third distinct genomovar within the species.

### Accession number(s).

This whole-genome shotgun project has been deposited at the European Nucleotide Archive (ENA) under the sequencing project number PRJEB25044. Scaffold sequences and annotations were deposited at DDBJ/EMBL/GenBank under the accession number OLKH00000000. The version described in this paper is the first version, OLKH01000000.
